# Detection of a Novel Metallo-β-Lactamase, CAM-2, in a Metagenome-Assembled Genome from China

**DOI:** 10.1128/spectrum.00261-22

**Published:** 2022-05-31

**Authors:** Rong Xiang, Meng Li

**Affiliations:** a Archaeal Biology Center, Institute for Advanced Study, Shenzhen University, Shenzhen, China; b Shenzhen Key Laboratory of Marine Microbiome Engineering, Institute for Advanced Study, Shenzhen University, Shenzhen, China; University of Guelph

**Keywords:** CAM-2, carbapenemase, metallo-β-lactamase

## LETTER

Carbapenems are regarded as important antimicrobials for the treatment of serious bacterial infections caused by multidrug-resistant (MDR) *Enterobacteriaceae*. In 2019, Boyd and colleagues from Canada (1) described a novel class B carbapenemase, named CAM-1, in the chromosome of four closely related clinical Pseudomonas aeruginosa isolates collected from three patients. No subsequent isolates harboring this gene have been reported. Recent studies showed that this gene belongs to the molecular subclass B1.4, a group dominated by *Bacteroidetes* and *Firmicutes* (2), suggesting that CAM-1 may have originated from a phylum other than *Proteobacteria*. Here, we report for the first time a novel metallo-β-lactamase, CAM-2, which is located on the chromosome of a metagenome-assembled genome (MAG) belonging to the family *Pyrinomonadaceae* (3).

In July 2016, three estuary sediment samples were collected at Shenzhen Bay, China. We then performed total DNA extraction from three sediment samples using a DNeasy PowerSoil kit (Qiagen, Germany). Metagenomic sequence data were generated using Illumina HiSeq sequencing with 150-bp paired-end reads at Novogene Bioinformatics Technology Co., Ltd. (Tianjin, China). Raw metagenomic reads were dereplicated (100% identity over 100% length) and trimmed using sickle (https://github.com/najoshi/sickle). The remaining high-quality metagenomic reads were *de novo* metagenome assembled using MEGAHIT (4) with the parameters “–min-contig-len 1000 –k-min 21 –k-max 141 –k-step 12 –merge-level 20,0.95” and filtering out contigs shorter than 1 kb. We used the ABRicate tool (v1.0.1) (https://github.com/tseemann/abricate) to identify antibiotic resistance genes present in our metagenomic assemblies. The ResFinder (5) database was used as the reference database for ABRicate with the following settings: a minimum nucleotide identity of 70 % and a minimum DNA coverage of 80 %. The results showed that the assembled contig sequences of one of the samples contained a *bla*_CAM_-like gene designated *bla*_CAM-2_, which was 741 bp long, 15 bases longer than the original *bla*_CAM-1_ gene (726 bp), and showed 91.21% nucleotide identity to *bla*_CAM-1_. In order to find out the bacterial host of the *bla*_CAM-2_ gene, genome binning of the assembled contigs was conducted using MetaBAT (6) with 12 sets of flags inducing different sensitivity and specificity combinations, and CheckM (7) was used to calculate the completeness and contamination of MAGs. Binning analysis produced a positive binning for the *bla*_CAM-2_ gene, consisting of 20 contigs (genome completeness, 94.84%; contamination, 3.42%). Finally, the *bla*_CAM-2_-carrying bin was selected for taxonomy classification using the GTDB-Tk package (8) and was taxonomically classified as the *Pyrinomonadaceae* family within the phylum *Acidobacteria* (3).

**FIG 1:**
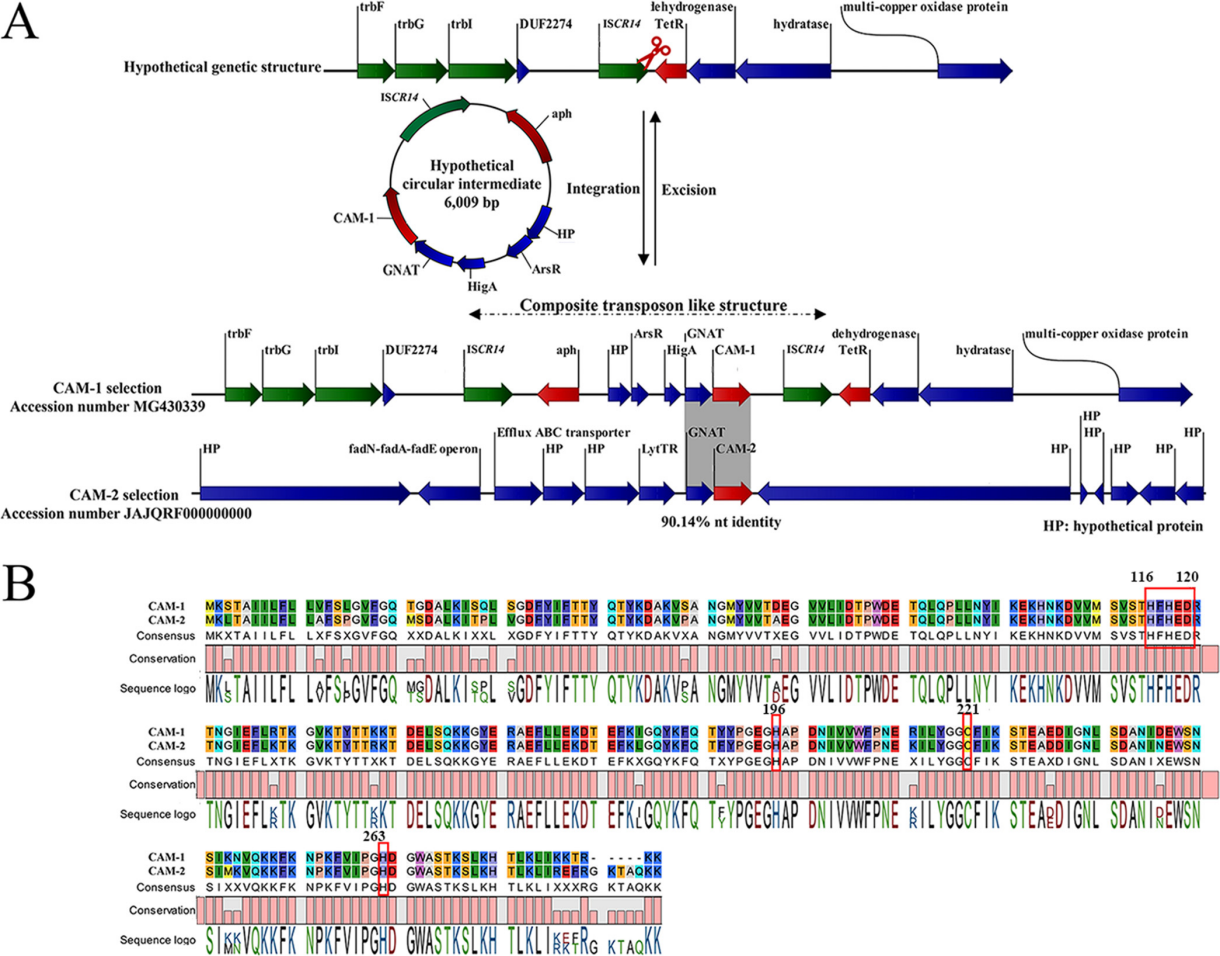
(A) Comparative analysis of the genetic environment of *bla*_CAM-1_ between the reference integrative mobilizable element IMEPaCAM-1 (accession no. MG430339) and the annotated metagenome contig; (B) alignment of the predicted amino acid sequences of CAM-1 and CAM-2.

The MAG consists of 5,719,470 bp. (The GC content is 46.4%.) The number of coding sequences (CDSs) is 5,465, as determined according to the Rapid Annotation Subsystem Technology (RAST) server (http://rast.nmpdr.org/). Except for *bla*_CAM-2_, this MAG does not carry other antibiotic resistance genes or mobile genomic elements. Sequence analysis revealed that *bla*_CAM-2_ was located on a contig of 1,194,427 bp with a GC content of 47.1%. Comparative analysis of the genetic environment of the *bla*_CAM-2_ gene, between the annotated metagenome contig and the reference ICEPaCAM-1 (GenBank no. MG430339), showed that a 1,233-bp segment, *bla*_CAM-2_-GNAT, exhibited 90.14% nucleotide identity to the corresponding region of ICEPaCAM-1. There was a 5,895-bp protein-coding sequence (CDS) downstream of *bla*_CAM-2_ in our contig instead of IS*CR14* in the reference sequence ICEPaCAM-1 (Fig. 1A). The *bla*_CAM-1_ gene was in a composite transposon-like structure flanked by two identical IS*CR14* elements. The IS*CR* elements have been implicated in mobilization of the adjacent sequences through rolling-cycle (RC) replication, during which circular intermediates will be generated (9). Therefore, based on the analysis of the structural features of ICEPaCAM-1, we propose a 6,009-bp circular intermediate may be involved in the formation of ICEPaCAM-1 (Fig. 1A). The GC content of P. aeruginosa chromosomal DNA ranges between 65 and 67% (10), whereas that of the *bla*_CAM-1_ gene is 43.8% and that of the *bla*_CAM-2_ gene is 44.8%, suggesting that *bla*_CAM_ did not originate from P. aeruginosa. The average chromosomal GC content of our MAG is 46.4%. The similar GC contents of *Pyrinomonadaceae* and the *bla*_CAM_ gene and the location of the *bla*_CAM-2_ gene on the chromosome of *Pyrinomonadaceae* in this study, as well as the fact that *bla*_CAM-2_ is not located within mobilizable element, indicate that *Pyrinomonadaceae* may be an ancestral source of the *bla*_CAM_ gene.

CAM-2 carried several missense mutations and was 5 amino acids longer than CAM-1, resulting in an amino acid sequence similarity of 90.46% to CAM-1 (Fig. 1B). Compared with CAM-1, CAM-2 had identical zinc ion-binding motifs: His116, His118, and His196 for Zn^1^ and Asp99, Cys177, and His219 for Zn^2^ (Fig. 1B). To confirm the role of this putative carbapenemase gene, the entire *bla*_CAM-2_ gene was synthesized at Beijing Tsingke Biotechnology Co., Ltd. (Beijing, China), and subcloned into the expression vector pET28a. Gene *bla*_CAM-2_ was amplified with two primers, the XhoI-containing primer CGGCAGCCATATGAAATTAACTGC as the forward primer and NdeI-containing primer GGTGGTGGTGCTCGAGCTATTTCTTTTGTGC as the reverse primer. PCR amplification was carried out with a GeneAmp PCR System 9700 in a total reaction volume of 20 μL under the following conditions: 96°C for 3 min, followed by 25 cycles of 15 s at 96°C, 15 s at 8°C, and 20 s at 72°C, and then a final extension of 3 min at 72°C. Both the PCR products and cloning vector pET28a (Tsingke, China) were digested with NdeI and XhoI restriction enzymes, and ligation was achieved using T4 DNA ligase (TaKaRa, China) according to the manufacturer’s protocol. The recombinant plasmid (pCAM-2) was transformed into Escherichia coli BL21 cells, selected on LB agar plates containing kanamycin (50 μg mL^−1^) and imipenem (1 μg mL^−1^), and incubated at 37°C for 16 h. We then picked single colonies from the LB agar plates and incubated the colonies in LB liquid medium, and then they were shaken at 250 rpm for 14 h at 37°C. Positive colonies were confirmed by restriction digestion and Sanger sequencing. To measure the MIC of the corresponding antibiotic in E. coli BL21 cells, liquid broth dilution tests were conducted as recommended by CLSI guidelines with Mueller-Hinton (MH) broth (Oxoid, United Kingdom). In general, bacterial cultures were diluted 100-fold in MH broth containing 2-fold dilutions of antibiotics ranging from 0.25 to 128 μg/mL and maintained at 37°C for over 20 h. E. coli BL21(DE3) strains carrying the pET28a vector with a synthesized CAM-1 gene insert or no insert were used as positive and negative controls, respectively. The E. coli pCAM-2 transformant exhibited the same resistance phenotype as CAM-1, with resistance to imipenem, meropenem, ampicillin, cefotaxime, ceftriaxone, ceftazidime, amoxicillin-clavulanic acid, cefoxitin, and piperacillin-tazobactam, but was susceptible to aztreonam (Table 1). The specific activity of the E. coli pCAM-2 transformant against imipenem was 1.83 mmol min^−1^ mg^−1^, as previously described (11).

**TABLE 1:** Antimicrobial drug susceptibility profile

Antibiotic	MIC (mg/L) for:		
	BL21/pET28a-CAM-1	BL21/pET28a-CAM-2	BL21/pET28a
Ampicillin	>128	>128	0.5
Cefotaxime	>128	>128	0.25
Ceftriaxone	>128	>128	0.25
Ceftazidime	>128	>128	0.5
Amoxicillin-clavulanic acid	>128	>128	0.25
Cefoxitin	>128	>128	1
Piperacillin-tazobactam	>128	>128	0.5
Imipenem	32	32	<0.25
Meropenem	8	16	<0.25
Aztreonam	0.25	0.25	0.25

To the best of our knowledge, this is the first time that the novel metallo-β-lactamase CAM-2 has been found in the family *Pyrinomonadaceae* in China. Since members of the family *Pyrinomonadaceae* have been isolated from soil environments (3), where they interact with bacteria from different sources, they may act as a reservoir for *bla*_CAM_ and contribute to its potential dissemination. The discovery of CAM-2 in the estuary environment means that CAM-2 may have spread to nearby hospital and other environments. Further studies are required to assess the prevalence of *bla*_CAM_ among *Enterobacterales* of clinical interest and to determine whether *bla*_CAM-2_ is a mobilizable antibiotic resistance.

### Data availability.

The metagenome-assembled genome generated and analyzed during the current study is available in NCBI and can be viewed under accession no. JAJQRF000000000.
